# A Modified Contact Angle Measurement Process to Suppress Oil Drop Spreading and Improve Precision

**DOI:** 10.3390/molecules27041195

**Published:** 2022-02-10

**Authors:** Xiao Deng, Xianmin Zhou, Muhammad Shahzad Kamal, Syed Muhammad Shakil Hussain, Mohamed Mahmoud, Shirish Patil

**Affiliations:** 1 Department of Petroleum Engineering, King Fahd University of Petroleum & Minerals, Dhahran 31261, Saudi Arabia; g201706030@kfupm.edu.sa (X.D.); mmahmoud@kfupm.edu.sa (M.M.); 2 Center for Integrative Petroleum Research, King Fahd University of Petroleum & Minerals, Dhahran 31261, Saudi Arabia; xzhou@kfupm.edu.sa (X.Z.); smshakil@kfupm.edu.sa (S.M.S.H.)

**Keywords:** contact angle, oil drop spreading, wettability, surfactant

## Abstract

Static contact angle measurement is a widely applied method for wettability assessment. Despite its convenience, it suffers from errors induced by contact angle hysteresis, material heterogeneity, and other factors. This paper discusses the oil drop spreading phenomenon that was frequently observed during contact angle measurements. Experimental tests showed that this phenomenon is closely related to surfactants in the surrounding phase, the remaining oil on the rock surface, and oil inside the surrounding phase. A modified contact angle measurement process was proposed. In the modified method, deionized water was used as the surrounding phase, and a rock surface cleaning step was added. Subsequent measurements showed a very low chance of oil drop spreading and improved precision. A further comparison study showed that, when the surrounding phase was deionized water, the measured contact angle values tended to be closer to intermediate-wet conditions compared to the values measured in clean surfactant solutions. This difference became more significant when the surface was strongly water-wet or strongly oil-wet. As a result, the developed process has two prerequisites: that the in-situ contact angle values inside surfactant solutions are not required, and that the wettability alteration induced by the surfactant solution is irreversible.

## 1. Introduction

Wettability is a description of the surface preference of a solid surface towards different fluids. In the petroleum industry, the wettability of formation rock is an important factor determining the types and amount of residual oil [1]. Widely used wettability assessment methods include contact angle measurement [2], the Amott–Harvey test, and USBM [3]. Other methods that are not so commonly used, including flotation test [4], chromatographic study [5], and NMR [6], also provide valuable knowledge about wetting states. Among all mentioned methods, contact angle measurement is the most applied method.

Contact angle (CA) measurement is a convenient method for wettability assessment [7,8,9,10,11]. It involves three phases: an inert solid phase and two immiscible fluids. The contact angle is usually observed between the tangent to the solid surface and the tangent to the fluids interface in the denser fluid [12]. In ideal conditions, the contact angle relates to the lowest Gibbs energy for the system [13]. However, this condition requires an isotropic, atomically flat, chemically non-reactive, rigid, and non-deformable surface [14]. Real conditions rarely meet such requirements. As a result, the apparent contact angle may instead relate to local minimal Gibbs energy [13]. The obtained contact angle values comprise a range of values. The highest contact angle value in the range is called the “advancing contact angle” and the lowest value is called the “receding contact angle”. The difference between advancing and receding contact angles is called “contact angle hysteresis” [15]. Studies suggest that the main causes of contact angle hysteresis are surface roughness and heterogeneity as well as metastable surface energetic states [16,17]. The CA value difference from repeated measurements on the same sample can be significant. For example, Seethepalli et al. found the difference between the largest and smallest CA values rose up to 62° [18]. Despite the convenience of static contact angle measurement, the significant hysteresis makes it difficult to precisely describe the wetting state or compare the wettability conditions of different surfaces.

Reducing the difference among values obtained from repeated CA measurements can help achieve a better assessment of wettability. Some studies focus on choosing the most reliable CA measurement technique(s). Kirk et al. applied statistical analysis for the selection of the CA measurement method. They concluded that the Wilhelmy-plate method obtained the greatest precision and sensitivity [19]. Bachmann et al. found the Wilhelmy-plate method and a modified capillary rise method to be reliable [20]. Some studies focus on improving the CA measurement technique(s). Vuckovac et al. found that a slight inconsistency between the detected baseline and the actual baseline could lead to around an 8° error for superhydrophobic surfaces. They suggested considering more precise force-based technologies for developing new methods [21]. Srinivasan et al. also worked on that issue and proposed a perturbation solution of the Bashforth-Adams equation as a more accurate method in estimating the contact angles of super-repellent surfaces [22]. Volpe et al. suggested applying acoustic vibration to help the interface move from metastable to a stable equilibrium state. In that way, there are higher chances of measuring the CA at the lowest Gibbs energy [23]. Konduru proposed a MATLAB-based program that automatically processes a batch of images, thus enabling the calculation of advancing and receding contact angle [24]. Some studies propose new methods or processes. Restagno et al. proposed a contact angle measurement technique based on the capillary bridge between a liquid and a spherical solid surface. They suggested this technique be particularly useful in surfaces with very low contact angle hysteresis [15]. Lamour et al. suggested a low-cost laboratory solution for CA measurement [25]. Korhonen et al. reported a procedure for accurate receding angle measurement. Their procedure emphasizes distinguishing the true receding movement from “pseudo movement” [26].

Many recent studies about static contact angle measurement focus on improving the image processing program. Stalder et al. proposed a snake-based imaging processing technique to improve the accuracy of contact angle measurement [27]. Heib and Schmitt developed a high-precision drop shape analysis method that focuses on the region near the contact point instead of the entire drop [14]. Guilizzoni proposed a contact angle image processing approach based on spline fitting and numerical integration [28]. Wen et al. developed a simple and computationally efficient contact angle obtaining method that utilized the lattice Boltzmann method [29]. Other related studies include the work of Atefi et al. [30], Chini and Amirfazli [31], Bateni et al. [32], Kalantarian et al. [33], Atae et al. [34], and many more.

This paper suggests that by modifying the CA measurement process, a frequently observed phenomenon, the oil drop spreading phenomenon, can be suppressed. A modified process is proposed. In Section 2.1, a statistical study on the preliminary CA value dataset showed high measurement error. In Section 2.2, tests were conducted to study the connection between rock surface cleanness, the surrounding phase, and the oil drop spreading phenomenon. Based on the results, a modified CA measurement process was proposed in Section 2.3. In Section 2.4, a statistical study on the CA value dataset obtained from measurements following the modified process was conducted. Section 2.5 compares results from preliminary dataset and secondary dataset. The comparison showed that by introducing a surface cleaning step and using deionized (DI) water as the surrounding phase, the oil drop spreading phenomenon was largely suppressed. An improvement in the precision of CA measurement was achieved. In Section 2.6, a further test about the effect of surrounding phase replacement was conducted. Results showed that changing the surrounding phase could induce a significant difference in the measured contact angle values, indicating that the modified process could only be applied when the in-situ CA values were not required.

## 2. Results and Discussion 

### 2.1. Preliminary Contact Angle Measurement Results

**Figure 1:**
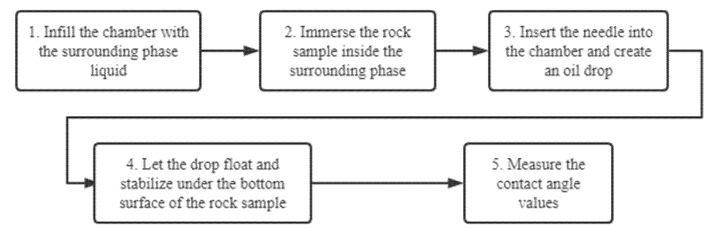
A schematic of the contact angle measurement process.

Indiana limestone samples were cleaned using toluene and heated for three days in the oven at 50 °C to remove the remaining toluene. Samples were then saturated with crude oil and oil-aged in the oven at 90 °C for more than 15 days. All of the oil-aged samples were then treated with surfactants solutions. Finally, CA measurements were conducted on the samples (Figure 1).

In the preliminary measurements, the process for CA measurement is arranged in this way:

1. Infill the liquid chamber with the surrounding liquid phase.
2. Immerse the rock sample inside the surrounding phase.
3. Insert the needle into the chamber and generate an oil drop.
4. Let the drop float and stabilize under the bottom surface of the rock sample.
5. Measure the contact angle values.

A batch of 331 samples was used. At least two measurements were conducted for each sample. After that, the average CA value and the corresponding standard deviation were calculated for each sample. Standard deviation (SD) was calculated to represent the measurement error. A dataset of 331 points, the preliminary dataset, was obtained.

**Figure 2:**
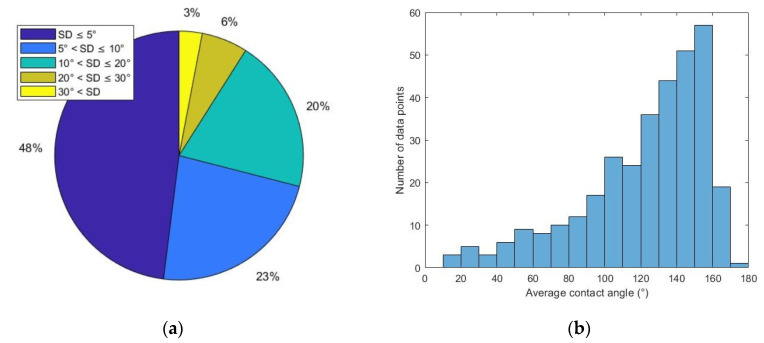
Statistical features of the preliminary dataset (**a**) percentage of each standard deviation range; (**b**) histogram of average contact angle.

Figure 2a is a pie chart showing the percentage of data with different SD ranges. A total of 161 points (48% of total) have SD less than 5°. A total of 75 points (23% of total) have SD values in the range of 5°~10°. A total of 66 points (20% of total) have SD values in the range of 10°~20°. A total of 20 points (6% of total) have SD values in the range of 20°~30°. Nine points (3% of total) have SD values in the range of 30°~60°.

Figure 2b shows that most of the CA values are in the range of 90°~180°. There are 53 points in the range of 0°~90° and 278 points in the range of 90°~180°. This unbalanced distribution can be explained by the experimental condition. Samples were oil-aged to oil-wet or strongly oil-wet conditions. Then, they were treated with surfactant solutions that have poor to moderate wettability alteration (WA) performance. Most of the surfactant-treated samples had intermediate-wet to oil-wet wettability after treatment. It is reasonable that the resulting contact angle values are more concentrated in the range of 90°~180°.

52% of the collected data has a standard deviation larger than 5°, indicating that the preliminary dataset has moderate to large error. Since the oil drop spreading phenomenon was frequently observed during measurements, a study on the connection between measurement error and the oil drop spreading phenomenon was conducted.

### 2.2. A Study on The Oil Drop Spreading Phenomenon

During the measurements, it was frequently observed that the oil drop spread on the rock surface. This phenomenon can indicate oil-wetness [35,36]. However, in our study, it was observed on samples that showed water-wetness. There seemed no or weak connection between the phenomenon and surface wettability. Besides, the oil drop could spread out till it became a thin oil film. It causes measurement difficulties or even failure due to the lack of an ellipsoidal droplet.

**Figure 3:**
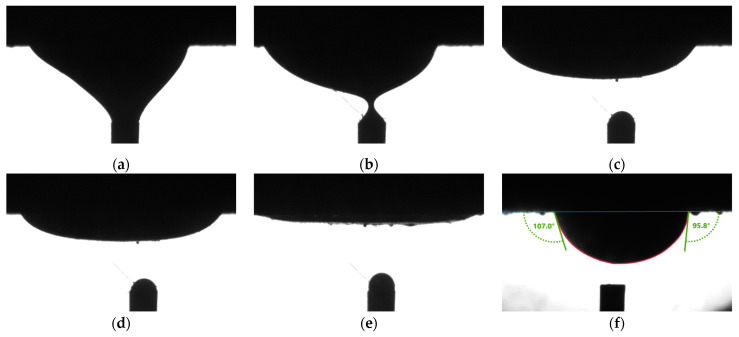
The oil drop spreading phenomenon observed in contact angle measurements (**a**) starting; (**b**) after 5 s; (**c**) after 15 s; (**d**) after 25 s; (**e**) after 10 min; (**f**) a drop that didn’t spread.

**Figure 4:**
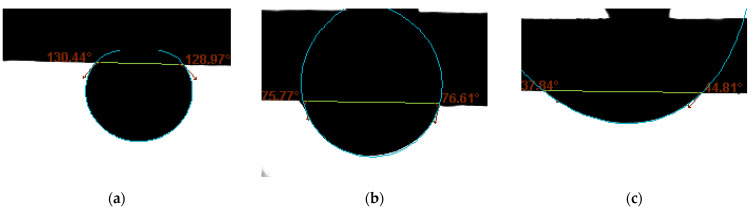
The ellipsoid shape of oil drops under different wetting conditions (**a**) water-wet; (**b**) intermediate-wet; (**c**) oil-wet.

Figure 3a to Figure 3c shows the spreading of an oil drop. The drop gradually diminished to a thin film in 10 min. On the same rock surface, another oil drop at a different spot didn’t spread. The CA values showed the intermediate-wet condition, as shown in Figure 3f. Figure 4 shows curvatures of oil drops in normal cases. Comparing Figure 3 and Figure 4, it is obvious that the curvatures of oil drops in Figure 3a–e are flatter.

The drop spreading phenomenon can be qualitatively defined with three levels. When the oil drop becomes slightly flattered without significant change in CA value, it is referred to as “slight spreading”. When the oil drop continues spreading to the extent that capturing CA values on both sides simultaneously became impossible, it is referred to as “severe spreading”. The level between “slight spreading” and “severe spreading”, in which CA values on both sides could still be captured simultaneously, is referred to as “moderate spreading” in the following context.

**Figure 5:**
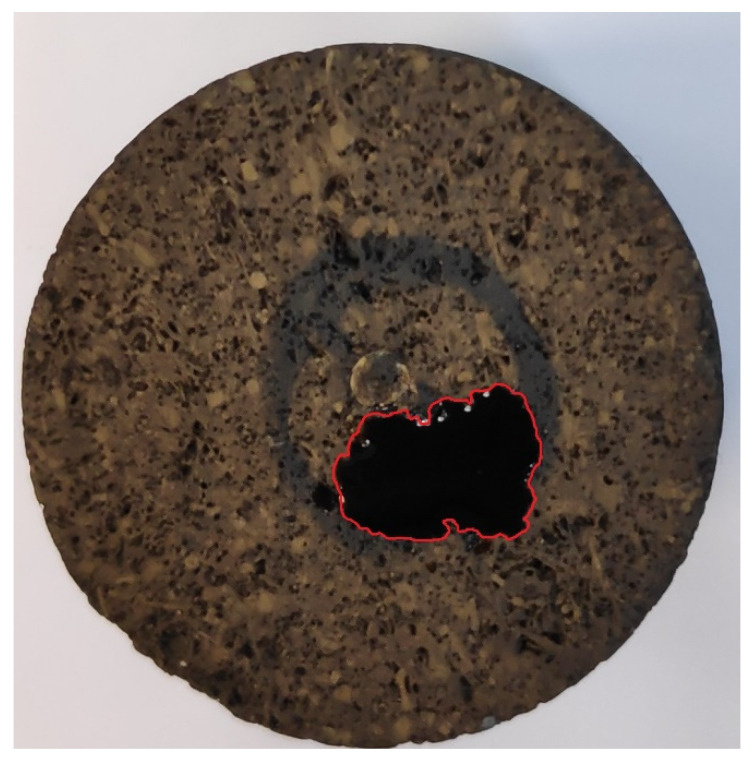
Irregular contact contour (in red).

Another feature related to the oil drop spreading phenomenon is the irregular contour of the drop. When moderate or severe spreading happened, the contact contour of oil drop became irregular. An artifact of the irregular contour is shown in Figure 5. On an ideal surface, the contact contour should be a circle. The irregular contour of oil drop indicates the rock surface heterogeneity, nonuniformity of the surrounding phase, or both.

Factors that influence CA measurements include sample heterogeneity [37,38], surface roughness [39,40], phase properties [41,42], temperature [43,44], pressure [45,46], humidity [47], electrical field [48], and others. Rock has some degree of heterogeneity by nature. However, Indiana limestone is usually regarded as relatively homogeneous [49]. XRD result shows that the samples have a mineral composition of 100% calcite. Besides, sample surfaces were smoothened in the same way, with sandpaper of 500cw. The same crude oil was used in all measurements. All of the measurements were carried out in the lab with a maintained room temperature of around 21 °C and stable humidity. The only factor that changed significantly among measurements was the surrounding phase properties, and the treatment time (which refers to the time of sample soakage inside surfactant solutions). Additionally, the measurement process could also have an impact.

(1) $$C_{s}=\sigma_{gw}-\sigma_{nw}-\sigma_{gn}$$

One possible reason for oil drop spreading could be the presence of surfactants. The definition of spreading coefficient, as given by Equation (1), emphasizes the impact of surface tension and interfacial tension (IFT) on the spreading phenomenon. In the equation, $C_{s}$ is the spreading coefficient. $\sigma_{gw}\;,\sigma_{nw}\;,\;\mathrm{and}\;\sigma_{gn}$ are the IFT between gas and the surface, nonaqueous liquid and the surface, gas and the nonaqueous liquid, respectively [50]. In our case, the surface is rock surface. Water and crude oil are the liquid phases on the surface. Surfactants reduce the IFT between phases and cause changes in the spreading coefficient. The oil drop spreading phenomenon is reported in studies regarding low IFT systems. Reed and Healy suggested that if spreading happens, the IFT value must be lower than $4\times 10^{-3}\;\mathrm{mN}/m\;$[51]. Njobuenwu et al. proposed an equation to describe the relationship between the radius of the spreading oil drop and IFT, in which lower IFT links with a higher oil drop radius [52]. Hirasaki and Zhang analyzed how the interplay of IFT and buoyancy affected the equatorial radius [53]. Their method of analysis can also be applied to the cases in our study. IFT maintained the ellipsoid contour. When surfactants were added, IFT was significantly reduced. As a result, the oil drop spread on the horizontal plane. 

Another possible cause relates to the remaining oil in the surrounding phase. The surfactant solutions were used as the WA treatment fluid and then used as the surrounding phase. During WA treatment, some oil phase dispersed into the surfactant solution. Consequently, the surfactant solutions contained some oil as an oil layer on top of the surfactant solution, as oil droplets inside the solution, or both. When there was an oil phase dispersed in the surfactant solution, the solution appeared brownish or “dirty”. The oil phase inside surfactant solutions may contaminate the rock surface and affect the spreading of oil drop.

The remaining oil on the rock surface could also affect the oil drop spreading phenomenon. The rock samples usually had some remaining oil phase distributed on the surface after WA treatment. When the generated oil drop contacted the rock surface, the remaining oil on the rock surface could contact the generated oil drop and affect the spreading of the oil drop.

Tests were conducted to study the influence of the remaining oil on rock samples and the surrounding phase. A type of surfactant with moderate WA performance was selected to prepare surfactant solutions. Measurements were conducted on 32 rock samples. Three different fluids, DI water, newly prepared surfactant solution (labeled as “Clean”), and surfactant solution used in the WA treatment (labeled as “Dirty”), were used as the surrounding phases. In some cases, rock samples were used for CA measurement right after WA treatment (labeled as “Dirty”). In other cases, rock samples were cleaned (labeled as “Clean”) by softly rubbing on paper to remove the remaining oil on rock surface before CA measurements. Results are listed in Table A1. Rows carrying the same sample number contain the measurement results from the same core sample. CA1, CA2, CA3, and CA4 represent values from repeated measurements on the same sample, after the same treatment, and at the same measurement condition. Apart from CA values, the qualitative extent of the spreading phenomenon was also recorded. 

To analyze the impact of oil drop spreading on CA values, Table 1 was made to compare the data obtained under different oil drop spreading conditions. On the same sample, the angles generally tended to be larger when moderate or severe spreading happened. For example, Sample 11, Sample 17, and Sample 19 showed an intermediate-wet state when slight or no spreading happened. However, when severe or moderate spreading happened, those samples showed an oil-wet state. In a word, the oil drop spreading phenomenon leads to deviated CA values that generally indicate stronger oil-wetness. 

Comparing the number of times when spreading happened, Table 2 is obtained. According to Table 2, when DI water was used as the surrounding phase, oil drop spreading happened more often on dirty rock surface than on clean rock surface, indicating that surface cleaning reduces the chance of oil drop spreading. When the rock surface was clean, oil drop spreading happened more often in clean surfactant solutions than in DI water, indicating that surfactants add to the chance of oil drop spreading. When surfactant solution containing oil was used as the surrounding phase, severe oil drop spreading phenomenon was more frequently observed than when clean surfactant solution was used, indicating that oil phase inside surfactant solution also adds to the chance of oil drop spreading. 

In summary, a few observations can be drawn about the oil drop spreading phenomenon:

1. The oil drop spreading phenomenon has a significant impact on static contact angle measurement by resulting in measurement failures or deviated CA values higher than the values measured without oil drop spreading.
2. The existence of surfactants in the surrounding phase largely increases the chance of oil drop spreading.
3. The existence of the oil phase inside the surfactant solution or on the rock surface adds to the chance of oil drop spreading.

### 2.3. Modification of Contact Angle Measurement Process

**Figure 6:**
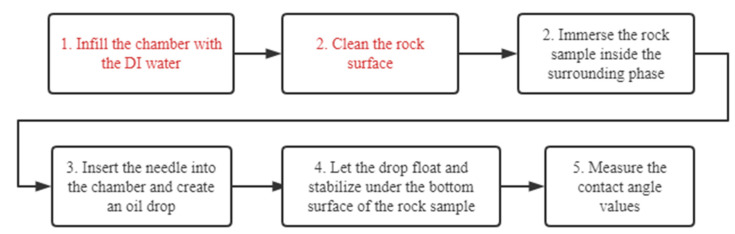
A schematic of the modified contact angle measurement process.

According to the observations from the previous section, we modified the CA measurement process to avoid oil drop spreading. The modified process is shown in Figure 6. The steps in red are additional or different compared to the unmodified process shown in Figure 1. The modified process is arranged in this way:

1. Infill the chamber with DI water.
2. Clean rock surface.
3. Immerse the rock sample inside the surrounding phase.
4. Insert the needle into the chamber and create an oil drop.
5. Let the drop float and stabilize under the bottom surface of the rock sample.
6. Measure the contact angle values.

The main features of the modified process are a surface cleaning step and using DI water as the surrounding phase. Rock sample surface cleaning was conducted by flushing the sample with DI water and softly rubbing the sample surfaces on a sheet of clean paper. Since samples had been treated with surfactant solutions, there would be remaining surfactant on sample surfaces. By flushing the samples with DI water, the remaining surfactant and some movable oil can be removed. As most of our tests were conducted at a relatively low surfactant concentration of 0.05 wt %, the time applied for flushing is about 10s. By softly rubbing the sample surfaces on a sheet of clean paper, the remaining oil is further removed. It is very important not to apply too much force during this step to avoid significant changes on the surface. We usually rub the surface of a sample two to three times softly. 

### 2.4. Secondary Contact Angle Measurement Results following the Modified Process

**Figure 7:**
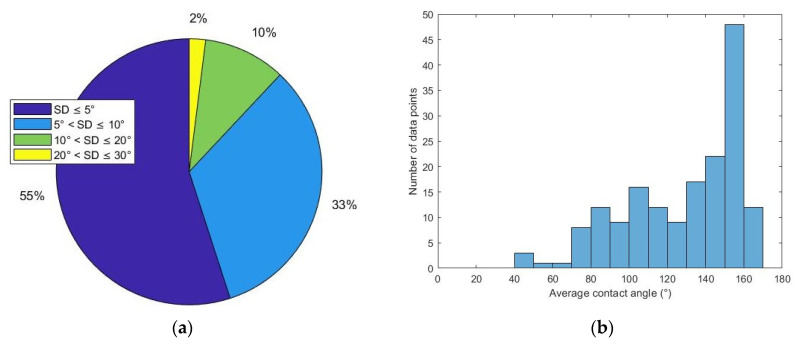
Statistical features of the secondary dataset (**a**) percentage of each standard deviation range; (**b**) histogram of average contact angle.

After conducting 340 measurements on 170 samples, a dataset of 170 average contact angle values was obtained. As shown in Figure 7, 93 points (55% of total) have SD less than 5°. Fifty-six points (33% of total) have SD values in the range of 5°~10°. Eighteen points (10% of total) have SD values in the range of 10°~20°. Three points (2% of total) have SD values in the range of 20°~30°. Although the secondary dataset contains about half the number of data points in the preliminary dataset, the distributions of the CA values appear similar. Most data points are in the CA range of 90°~180°. 

### 2.5. Comparison between Preliminary and Secondary Measurement Results

Table 3 compares the percentage of each SD range of both datasets. The modified process exhibited significant improvement in precision which came mainly from data of moderate error level (23% vs. 33%).

Both preliminary and secondary datasets are collections of the CA values obtained in different conditions. The varying conditions include surfactant type, surfactant concentration, solution volume, treatment time, and brine composition. With so many varying conditions, the significant improvement may not be solely attributed to the process modification. To further confirm that the modification helps reduce error, several detailed comparison analyses were conducted.

**Figure 8:**
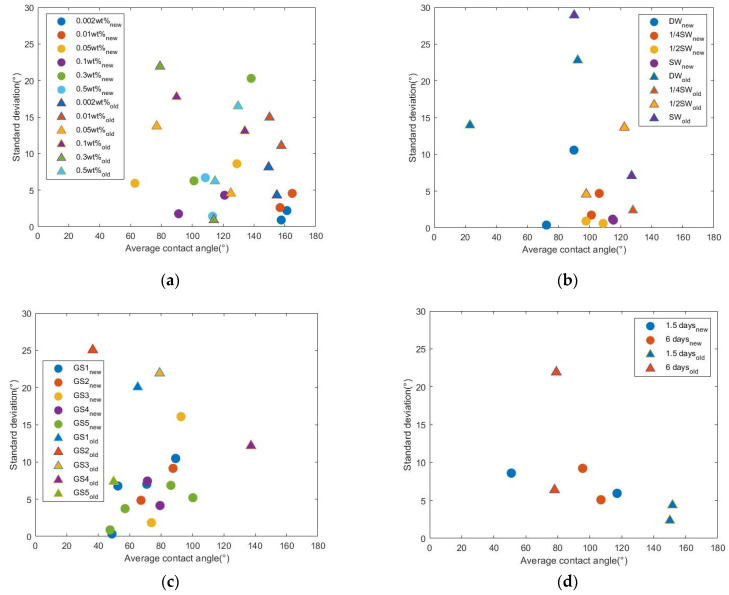
The comparisons of data obtained following unmodified and modified processes with the other changing condition as (**a**) different concentrations of GS3; (**b**) different salinity (SW, synthetic seawater; 1/2SW, 1/2 dilution of SW; 1/4SW, 1/4 dilution of SW; DW, deionized water. Surfactant GS3 of 0.05 wt % was applied); (**c**) different surfactants (GS1~GS5 of 0.05 wt %); (**d**) different treatment time.

Four data subsets are selected from the preliminary and secondary datasets. Surfactant solutions of the same surfactant GS3, but different concentrations, were prepared and used for the wettability alteration on oil-aged limestone samples. Two batches of rock samples were used. Both batches were divided into six groups. Each group had two samples. In the same batch, different groups of samples were treated with surfactant solutions with different concentrations. After the treatment, two CA measurements were conducted on each sample. Figure 8a shows the distribution of SD over average CA of all samples. The measurements of Batch 1 followed the modified process (labeled ‘new’). The measurement of Batch 2 followed the old process (labeled ‘old’). Data points of samples treated with surfactant solutions of the same concentration have the same color but different shapes. For example, the first groups in Batch 1 and Batch 2 were treated with GS3 solution of the same concentration (0.002 wt %). The data points of the first group in Batch 1 are shown by blue dots. The data points of the first group in Batch 2 are shown by blue triangles. In the figure, each dot or triangle represents one sample. Regardless of the surfactant concentration, most dots are lower than triangles of the same color, which indicates that measurements followed the modified process has a lower error level. For example, the red dots lower than the red triangles, suggesting that the error level of the red dots is lower than that of the red triangles. This means when all of the conditions are kept the same, measurements following the modified process have a lower error level.

The other three figures in Figure 8 are plotted in the same way as Figure 8a. Figure 8b compares the results from measurements where the changing conditions were the salinity of the surfactant solutions and the measurement process. The surfactant solutions were prepared by dissolving surfactants in diluted synthetic seawater. In Figure 8c the changing conditions were the surfactant type and the measurement process. In Figure 8d the changing conditions were the surfactant treatment time and the measurement process. In these figures, data of the same salinity, or surfactant type, or treatment time, show the improved precision brought by the modified process, such as what is concluded from Figure 8a.

When all of the other conditions are kept the same between two batches, a lower error level is repeatedly observed in the measurements from Batch 1 that followed the modified process. These analyses confirmed that the suggested process modification can improve measurement precision.

Another important observation from these figures indicates the limitation of the modified process. When all of the other conditions are kept the same, the average CA values from both batches appear to be close in many cases. For example, in Figure 8a, the two average CA values of red dots are close to 160°. The two average values of red triangles are close to 152°. This observation suggests consistency between the measurements following two different processes. However, this consistency does not apply to all data in the figures. For example, Figure 8d shows a large difference in average CA values between two batches. The possible difference brought by the modified process is discussed in the following section.

### 2.6. Limitation of The Modified Process

Using DI water as the surrounding phase has several advantages: (1) it reduces the chance of oil drop spreading; (2) it keeps IFT constant. When IFT is reduced, the volume of oil drop generated will reduce. Researchers have observed that changes in oil drop volume will lead to changes in CA value [16,54]. Some studies highlight the influence of the oil drop volume. Korhonen et al. suggested a “surprisingly large” initial drop size to be necessary to achieve reliable receding angle results [26]. If CA values are measured inside surfactant solutions, the interpretation from CA results to wettability will have to consider differences in IFT and drop size. Using DI water as the surrounding phase ensures a relatively constant oil drop volume. The CA value difference before and after WA treatment can be regarded as the “net wettability change” that minimizes the influences of IFT change or drop size change. 

On the other hand, the change of the surrounding phase may affect rock wettability. For cationic surfactants, the main WA mechanism is reported to be taking away the negatively charged organic materials adsorbed on the rock surface by ion-pairing [55]. In that case, the WA is irreversible. Using DI water as the surrounding phase will not change the rock wettability. When surfactants of reversible WA effect are used, such as anionic surfactants that change wettability mainly by adsorbing onto a rock surface, using DI water as the surrounding phase can wash away part of the surfactant molecules on rock surface, resulting in a changed wettability of rock.

Although the modified process showed improved precision, the change of surrounding phase from surfactant solution to DI water makes a different three-phase system. In the study or simulation of oil displacement, contact angle is involved in the calculation of important parameters such as work of adhesion, capillary pressure, the bond number, the trapping number and so on. If the contact angle value changes with the surrounding phase, it will be necessary to use surfactant solution as the surrounding phase when the in-situ CA values are needed.

A test was conducted to study how changing the surrounding phase affects the CA values. Measurements on 43 samples were conducted inside clean surfactant solutions. After that, measurements were repeated inside DI water. Table A2 compiles the averaged CA values obtained inside DI water and inside clean surfactant solutions. The CA difference is calculated by deducting the angle measured in DI water from the angle measured in surfactant solutions. The CA difference ranges from 0.3° to 46.3°. The average difference between CA values is about −6.2°.

**Figure 9:**
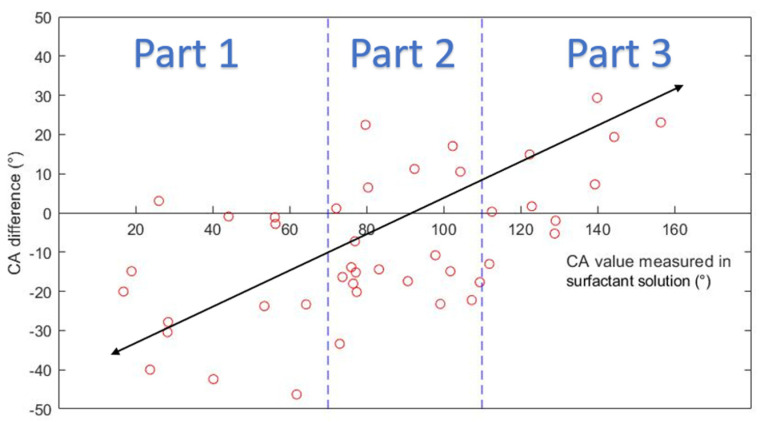
Contact angle difference vs contact angle measured in surfactant solutions.

Figure 9 plots the CA difference along the CA. The figure can be divided into three parts:

Part 1: in the range of 0°~70°, most of the CA values measured in DI water were larger than those measured in surfactant solutions. When CA values in surfactant solutions indicate water-wet condition, CA values in DI water indicate less water-wet or intermediate-wet condition.

Part 2: in the range of 70°~110°, CA values measured in DI water could be larger or smaller than those measured in surfactant solutions.

Part 3: in the range 110°~180°, most of the CA values measured in DI water were smaller than those measured in surfactant solutions. When CA values in surfactant solutions indicate oil-wet condition, CA values in DI water indicate less oil-wet or intermediate-wet condition.

Results show that changing the surrounding phase from surfactant solutions to DI water can cause a significant difference in the measured CA values, especially when the surface is strongly water-wet or strongly oil-wet. The significance of the difference is related to the wetting state of the surface. Based on the result, a conclusion can be drawn that DI water should not be used as the surrounding phase when in-situ CA values are needed.

A similar comparison study is conducted regarding the advancing and receding contact angle. In this study, the advancing and receding contact angle was measured by expanding and decreasing the oil droplet size. Similar measurement difficulty induced by the oil drop spreading was observed.

The comparison study was conducted on two rock samples. Advancing and receding contact angle values were measured in surfactant solution and in DI water. Results are listed in Table 4. The rock sample saturated with DI water had an initial wettability condition of strongly water-wet. The surrounding phase seemed to have a negligible impact on the advancing and receding angles. The other rock sample was oil aged and then treated with a surfactant solution. There was a difference of 35.2° between the advancing angle, indicating that changing the surrounding phase can lead to significant changes in the advancing angle value.

**Figure 10:**
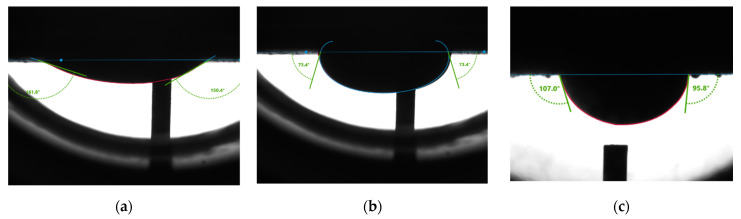
Contact angle measured inside surfactant solution (**a**) receding angle; (**b**) advancing angle; (**c**) static contact angle.

Figure 10 shows measurement results from the second rock sample. The average receding angle (Figure 10a) is 156.1°. The average advancing angle (Figure 10b) is 73.4°. Figure 10c showed an average water contact angle of 101.4°, indicating intermediate wetness of the rock surface.

In a word, the modified process may not apply to all conditions. There are two prerequisites for applying the modified process:

1. The in-situ CA value inside the surfactant solution is not required.
2. The wettability alteration effect of surfactant solution is irreversible.

If either of the prerequisites is not satisfied, this modified process is not applicable. In that case, either different contact angle measurement techniques or other wettability assessment methods should be applied. One alternative method is to relate the spreading phenomenon with surface wettability and other interfacial properties. For example, Chen related the drop spreading process with dynamic contact angle when the capillary and viscous forces are the only dominant factors [56]. Lavi and Marmur proposed an empirically derived equation that matched the drop spreading phenomenon very well [57]. Chen and Bonaccurso showed that the power-law developed for the description of the dynamic wetting process has an exponent that is dependent only on the surface wettability [58].

Another limitation of this modified process is that it is based on static contact angle measurement which cannot ensure reaching the lowest Gibbs energy condition. As a result, contact angle hysteresis still introduces error to the measurement results. 

## 3. Materials & Methods

Filtered crude oil (API 31.07, viscosity 12.492 cP, density 0.87 g/mL at 25 °C) and Indiana limestone core plugs of porosity 14.6% were used in this study. XRD results suggest that the Indiana limestone core has a mineral composition of 100% calcite. Core plugs were cored and cut into slices of 1-inch diameter and around 3~4 mm thickness. After that, the sandpaper of particle size P500 was used to smooth their surfaces.

A total of 14 kinds of locally synthesized ethoxylated quaternary ammonium gemini surfactants [59,60] were used in this study. These surfactants have the same head groups and tail groups. The only two differences among them are the structure of spacer, and counter-ions. Table 5 summarizes the structure of tested surfactants. They have shown advantages in interfacial properties [61], clay swelling inhibition [62], and foaming properties [63]. The concentration of the surfactant solutions varied from 0.002 to 0.50 wt %, mostly 0.05 wt %. The IFT values in DI water at CMC are around the magnitude of $1\times 10^{-2}\;\mathrm{mN}/m$ [61]. The IFT between crude oil and the surfactant solution is measured by the spinning drop method.

**Figure 11:**
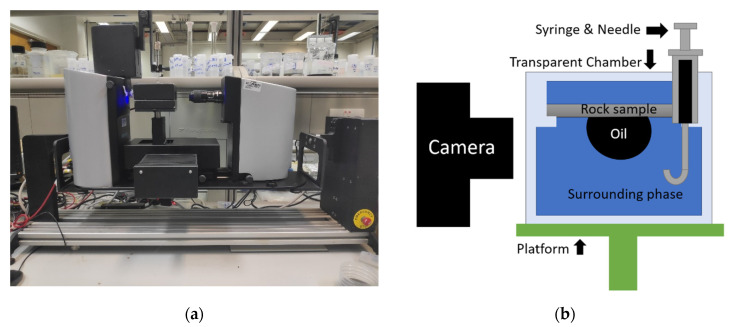
Contact angle measurement method (**a**) the CA measurement instrument; (**b**) an illustration of how oil/water CA is measured.

Static contact angle measurements were conducted using the Attension Tensiometer provided by Biolin scientific, as shown in Figure 11. This device consists of a platform movement control system, a computer imaging system, and a syringe holder. A transparent chamber containing a rock substrate within the surrounding liquid phase is mounted onto the platform adjusted horizontally. The syringe with a hooked needle (outer diameter 0.734 mm) to create oil drops is installed onto the syringe holder. An oil drop is made from the tip of the needle. Due to the buoyancy caused by the density difference between oil and the aqueous phase, the oil drop leaves the needle tip and floats upward until contacting the rock sample bottom surface. After the shape of the oil, drop stabilizes, contact angle measurements are conducted. The volume of the transparent liquid chamber is around 60 mL. All measurements were conducted at room temperature of around 21 °C and atmosphere pressure. In the surfactant solution, the size of the oil drop was around 15 µL. In DI water, the size of the oil drop was around 27 µL.

Advancing and receding contact angles were measured by expanding and decreasing the oil drop size in the drop shape analyzer provided by Kruss. The needle has an inner diameter of 0.50 mm and an outer diameter of 0.843 mm. Measurements were conducted at room temperature (about 21 °C) and atmospheric pressure. The chamber can contain around 15 mL of liquid.

## 4. Conclusions

An observed phenomenon, oil drop spreading, is studied regarding its causes and influence on measurement results. Previous studies focus on the effect of low IFT on oil drop spreading. In this paper, two other causes of oil drop spreading were found. Several conclusions around the oil drop spreading phenomenon are drawn:

1. Oil drop spreading phenomenon results in measurement failure or increased error in contact angle values. The existence of surfactants in the surrounding phase is the main factor that induces oil drop spreading. The existence of the remaining oil phase on the rock surface or inside the surrounding phase also increases the possibility of oil drop spreading.
2. By adding a sample surface cleaning step and changing the surrounding phase from surfactant solutions to DI water, the process modification suppressed the oil drop spreading and improving the measurement precision. The improvement mainly came from the increase in the percentage of moderate error level (5° < SD ≤ 10°) data and a decrease in the percentage of high error level (SD > 10°) data.
3. Changing the surrounding phase from surfactant solutions to DI water can cause a significant CA difference up to 46.30° in the measured CA values. The significance of the difference is related to the wetting state of the surface. The modified process does not apply to measurements where in-situ CA values of surfactant solution/oil/rock system is required, or the wettability alteration is reversible.

## Figures and Tables

**Table 1:** Comparison of Contact Angle Values Obtained under Different Oil Drop Spreading Conditions.

Sample No.	Severe or Moderate Spreading CA data (°)	Average CA (°)	Slight or No Spreading CA Data (°)	Average CA (°)
3	98.1	98.1	77.4, 102.2, 89.1, 95.9, 94.9	91.9
5	162.0, 159.5, 128.9	150.1	134.2	134.2
9	131.04	131.0	119.3, 133.8	126.6
11	150.0, 152.4, 137.5, 142.4	145.6	100.9, 120.3	110.6
12	138.0, 150.7	144.4	151.7, 134.0, 128.2, 112.7	131.7
13	159.3, 158.5, 135.0, 123.2	144.0	124.2, 138.00	131.1
14	108.7	108.7	132.8, 134.5, 112.0, 102.9, 136.1	123.7
17	142.6, 143.3, 156.4	147.4	95.1, 75.6	85.4
19	169.3	169.3	81.6, 67.7	74.7
21	106.1, 123.4	114.8	101.7	101.7

**Table 2:** Oil Drop Spreading Phenomenon in Different Cases.

Material Properties		Spreading Phenomenon				Total Measurements
Rock	Surrounding Phase	Severe	Moderate	Slight	No	
Clean	DI	0	1	5	42	48
Dirty	DI	4	3	3	14	24
Clean	Clean	8	12	2	8	30
Dirty	Clean	2	5	0	3	10
Clean	Dirty	4	1	0	0	5
Dirty	Dirty	9	1	0	0	10

**Table 3:** A Comparison of Standard Deviation Distribution in Preliminary and Secondary Contact Angle Measurement Results.

Dataset	CA Range	SD ≤ 5°	5° < SD ≤ 10°	10° < SD ≤ 20°	20° < SD ≤ 30°	30° < SD
Preliminary (443)	0°~180°	48%	23%	20%	6%	3%
Secondary (170)	0°~180°	55%	33%	10%	2%	0%

**Table 4:** Advancing and Receding Contact Angle of Two Samples Obtained in Different Surrounding Phases.

Sample	Angle Measured in Surfactant Solution		Angle Measured in DI Water	
	Advancing	Receding	Advancing	Receding
Saturated with DI water	61.4°	14.0°	63.9°	7.1°
Oil aged and treated with surfactant solution	156.1°	73.4°	120.9°	76.9°

**Table 5:** The Molecular Structure of Applied Surfactants.

Name	Structure
GS1	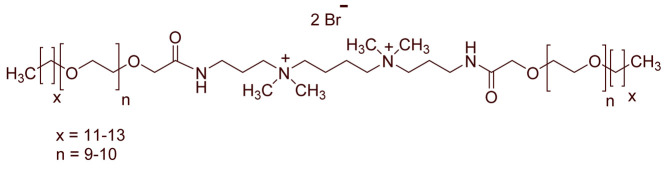
GS2	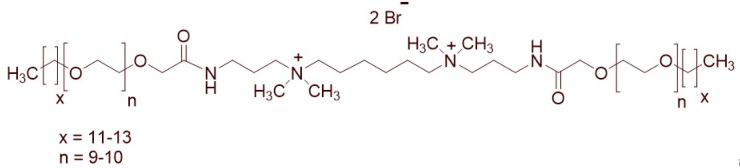
GS3	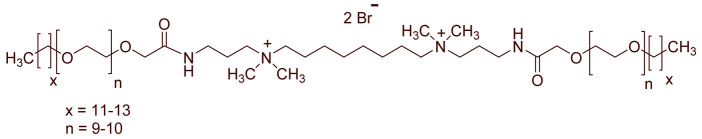
GS4	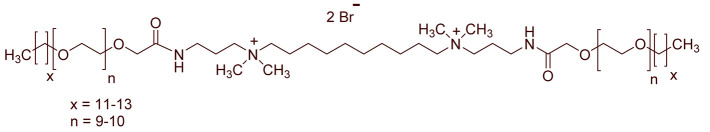
GS5	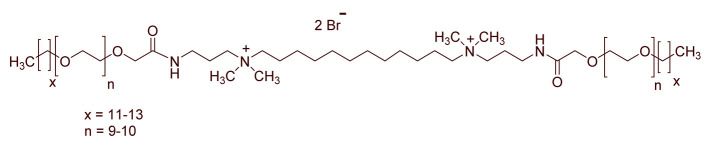
GS6	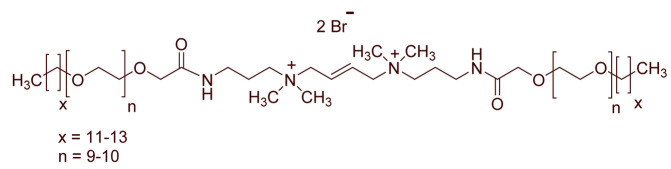
GS7	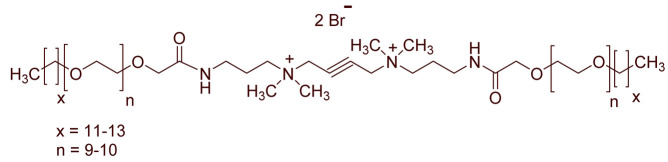
GS8	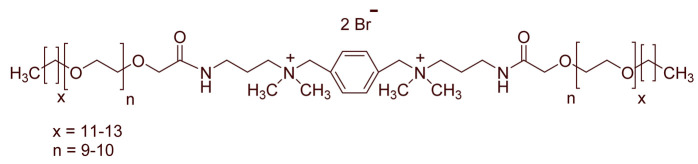
GS9	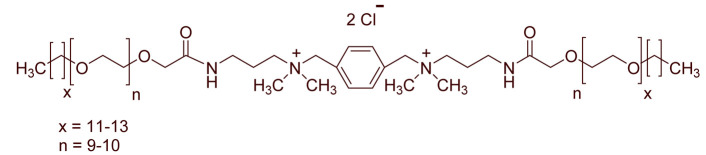
GS10	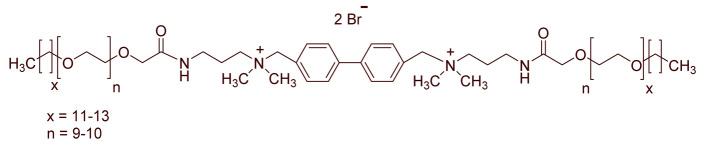
GS11	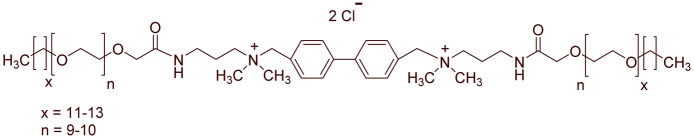
GS-NH	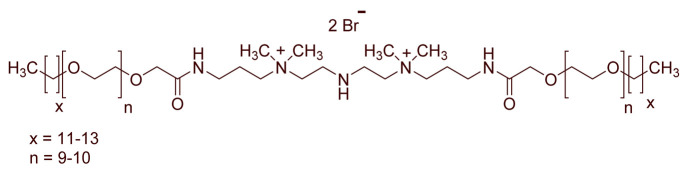
GS-OH	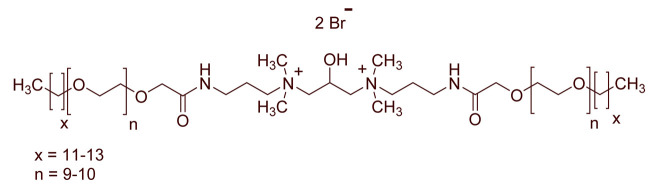
GS-EO	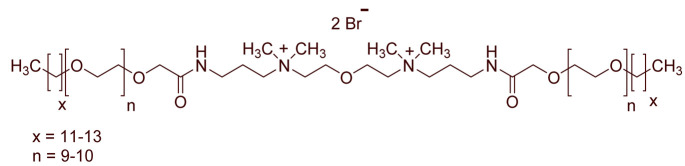

## Data Availability

The data presented in this study are available on request from the corresponding author.

## Sample Availability

Samples of the cationic gemini surfactants are available from the authors.

## Appendix A. Record of the Test on the Factors of the Oil Drop Spreading Phenomenon

Two factors were considered: (1) remaining oil on the rock samples; and (2) the remaining oil in the surrounding phase. Contact angle measurements were conducted under different sample cleaning and surrounding phase conditions. All results were organized in Table A1. If the contact angle value was obtained during the measurement, it was then recorded. If the measurement failed and no contact angle value was obtained, a “Failed” was recorded. An assessment of the spreading severity was made during each measurement and was recorded.

**Table A1:** A Study of the Influence of The Remaining Oil and The Surrounding Phase on Oil Drop Spreading Phenomenon.

Sample No.	Sample Cleaning	Surrounding Phase	Spreading	CA1 (°)	CA2 (°)	CA3 (°)	CA4 (°)	SD (°)	Average CA (°)
1	No	Dirty	Severe	Failed					
1	No	DI	No	103.8					103.8
1	Yes	DI	No	81.3					81.3
1	Yes	Clean	No	92.5					92.5
2	No	Dirty	Severe	Failed					
2	Yes	DI	No	122.8					122.8
2	Yes	Clean	No	62.1	82.2			10.1	72.2
2	Yes	DI	No	71.0					71.0
3	Yes	Dirty	Moderate	98.1					98.1
3	Yes	Clean	No	77.4	89.1			5.8	83.3
3	Yes	DI	No	102.2	95.9	94.9		3.2	97.7
4	No	Clean	No	97.1	117.5			10.2	107.3
4	No	DI	No	122.6	136.6			7.0	129.6
4	Yes	DI	No	109.2					109.2
4	Yes	DI	No	106.2	108.8			1.3	107.5
5	No	Dirty	Moderate	162.0					162.0
5	No	Clean	Moderate	159.5					159.5
5	Yes	DI	No	134.2					134.2
5	Yes	Clean	Severe, moderate	Failed	128.9				128.9
6	Yes	DI	No	93.5	61.0			16.3	77.3
6	Yes	Clean	No	70.9	36.0			17.5	53.5
7	No	Dirty	Severe	Failed					
7	No	Clean	Severe	Failed					
7	Yes	Clean	Severe	Failed					
7	Yes	DI	No	96.7					96.7
8	No	DI	No	121.4	133.3			6.0	127.4
8	Yes	Clean	Severe	Failed	Failed	Failed			
8	Yes	DI	No	116.3	135.6			9.7	126.0
9	No	DI	Moderate, Severe	131.0	Failed				131.0
9	Yes	DI	No	119.3	133.8			7.3	126.6
9	Yes	Clean	Severe	Failed	Failed				
10	No	DI	No	107.2	98.4			4.4	102.8
10	Yes	DI	No	93.5	95.6			1.1	94.6
10	Yes	Clean	No, Severe	76.5	Failed				76.5
11	No	DI	Moderate	150.0	152.4			1.2	151.2
11	Yes	DI	No	100.9	120.3			9.7	110.6
11	Yes	Clean	Moderate	137.5	142.4			2.5	140.0
12	No	DI	Severe, No	Failed	151.7				151.71
12	Yes	DI	No	134.0	128.2	112.7		9.0	125.0
12	Yes	Clean	Moderate	138.0	150.7			6.3	144.4
13	No	DI	Severe	159.3	158.5			0.4	158.9
13	Yes	DI	No	124.2	138.0			6.9	131.1
13	Yes	Clean	Moderate	135.0	123.2			5.9	129.1
14	No	DI	little	132.8	134.5			0.8	133.7
14	Yes	DI	No	112.0	102.9			4.6	107.5
14	Yes	Clean	Moderate, No	136.1	108.7			13.7	122.4
15	No	Dirty	Severe	Failed	Failed	Failed			
15	No	Clean	Moderate twice; severe once	80.8	Failed	121.6		20.4	101.2
16	No	Dirty	Severe	Failed					
16	Yes	DI	Slight	110.1	109.6			0.3	109.9
17	No	Dirty	Severe	142.6	143.3			0.4	143.0
17	Yes	Dirty	Severe	156.4					156.4
17	Yes	DI	Slight; no	95.1	75.6			9.8	85.4
18	Yes	Dirty	Severe	Failed	Failed				
18	Yes	DI	No	55.5	79.3			11.9	67.4
19	Yes	Dirty	Severe	169.3					169.3
19	Yes	DI	No	81.6	67.7			7.0	74.7
20	No	DI	No	95.5	103.4	106.6	101.5	4.0	101.8
20	Yes	Clean	Slight	102.6	103.7			0.6	103.2
21	Yes	DI	No	101.7					101.7
21	Yes	Clean	Moderate	106.1	123.4			8.7	114.8
22	Yes	DI	No	133.8	121.4			6.2	127.6
23	Yes	DI	No	141.1	143.9			1.4	142.5
24	Yes	DI	No	130.3	117.3			6.5	123.8
25	No	Clean	No	96.4	120.4			12.0	108.4
26	Yes	Clean	Moderate	101.5	109.8			4.2	105.7
27	No	Clean	Moderate	103.6	105.1			0.8	104.4
28	No	DI	No	152.6					152.6
29	Yes	DI	Moderate; no	125.8	118.4			3.7	122.1
30	Yes	DI	No	109.8	121.3			5.8	115.6
31	No	DI	Slight; no	141.7	145.8			2.1	143.8
32	Yes	DI	Slight	91.0	107.5			8.3	99.3

## Appendix B. Difference between the Contact Angle Values Obtained in DI Water and in Clean Surfactant Solutions

Replacing the surrounding phase with deionized water results in a different solid/liquid system. To study its influence on the measurement results, the contact angle values measured in surfactant solution and in deionized water of the same core were obtained and compared in Table A2.

**Table A2:** Comparison Study between CA Values Measured in Surfactant Solution and DI Water.

	CA in Surfactant Solution (°)	CA in DI Water (°)	Difference (°)	Test No.	CA in Surfactant Solution (°)	CA in DI Water (°)	Difference (°)
1	28.3	58.8	−30.5	23	101.8	116.7	−14.9
2	104.4	93.9	10.5	24	61.9	108.2	−46.3
3	40.2	82.6	−42.4	25	19.0	33.9	−14.9
4	28.5	56.3	−27.8	26	44.2	45.1	−0.9
5	156.5	133.4	23.1	27	90.7	108.1	−17.4
6	79.8	57.3	22.5	28	23.8	63.8	−40
7	64.3	87.7	−23.4	29	97.9	108.7	−10.8
8	112.6	112.3	0.3	30	16.9	36.9	−20
9	139.4	132.1	7.3	31	111.9	124.9	−13
10	56.4	59.2	−2.8	32	56.2	57.3	−1.1
11	122.9	121.3	1.6	33	77.5	97.6	−20.1
12	73.1	106.4	−33.3	34	80.4	74.0	6.4
13	109.4	127.1	−17.7	35	99.2	122.4	−23.2
14	77.2	92.4	−15.2	36	77.0	84.2	−7.2
15	73.8	90.1	−16.3	37	76.1	89.9	−13.8
16	26.1	23.0	3.1	38	102.4	85.4	17
17	92.5	81.3	11.2	39	76.5	94.6	−18.1
18	72.2	71.0	1.2	40	139.9	110.6	29.3
19	83.2	97.7	−14.5	41	144.3	125.0	19.3
20	107.3	129.6	−22.3	42	129.1	131.1	−2
21	128.9	134.2	−5.3	43	122.4	107.5	14.9
22	53.5	77.2	−23.7				
